# Generalized q-Method Relative Pose Estimation for UAVs with Onboard Sensor Measurements

**DOI:** 10.3390/s25061939

**Published:** 2025-03-20

**Authors:** Kyl Stanfield, Ahmad Bani Younes, Mohammad Hayajneh

**Affiliations:** 1 Guidance, Navigation, & Controls (GNC) Engineer, 5500 Campanile Drive, San Diego, CA 92182, USA; kylstanfield@sbcglobal.net; 2 Department of Aerospace Engineering, San Diego State University, San Diego, CA 92181, USA; 3 Faculty of Engineering, Mechatronics Engineering, The Hashemite University, Zarqa 13133, Jordan; mhayajneh@hu.edu.jo

**Keywords:** q-method, drone, UAV, pose, estimation, attitude, quaternion, relative, navigation

## Abstract

The q-method for pose estimation utilizes on-board measurement vectors of reference objects to calculate air vehicle position and orientation with respect to an Inertial frame. This new method solves for the quaternion eigenvalue solution of the optimal pose to minimize the error in the derived system of equations. The generalized q-method extends Davenport’s q-method for satellite attitude estimation by incorporating inertial position into the relative model and eliminating assumptions throughout the derivation that require spacecraft applications. Thus, the pose estimation model is developed and implemented for UAV applications using an onboard camera to obtain measurements in a controlled environment. Combined with numerical methods, algorithm outputs for position and orientation are validated against truth data to prove accurate estimation despite sensor error.

## 1. Introduction

In aerospace engineering and robotics, the estimation of attitude and position is an essential factor for precise autonomous operations [1,2,3,4,5]. Attitude estimation enables one to determine the vehicle’s orientation in a three-dimensional space, while position estimation determines the vehicle’s translational coordinates in a three-dimensional space. Modern methods integrate optimal filtering techniques by fusing measurements from local or global sensors with knowledge of the vehicle’s motion dynamics. Traditional methods often involve sensor fusion techniques combining visual, inertial, and GPS data. Visual odometry and simultaneous localization and mapping (SLAM) have been widely adopted for pose estimation using cameras and other imaging sensors, while inertial measurement units (IMUs) are often used to provide complementary motion information [6]. Techniques such as the Kalman filter, Extended Kalman Filter (EKF), and Unscented Kalman Filter (UKF) have been extensively applied. For instance, Zhang et al. [7] demonstrated the effectiveness of EKF in fusing data from inertial measurement units (IMUs) and global navigation satellite systems (GNSSs) to improve the robustness of UAV positioning in urban environments. Similarly, Liu et al. [8] explored the use of particle filters for attitude estimation in multi-sensor setups, highlighting their resilience to sensor noise and dynamic changes. One significant challenge in these methods is to deal with sensor noise and environmental conditions [9] that affect sensor performance, such as poor lighting or featureless scenes. Several researchers have focused on enhancing these techniques through the integration of LiDAR and deep learning-based approaches [10] to improve robustness and real-time performance, particularly in GPS-denied environments. Another challenge is associated with handling the coupled dynamics with the estimated position and attitude simultaneously [11]. Therefore, this research develops a generalized formulation that combines the estimation of position and attitude based on the well-known q-method framework [12].

Using the quaternion (q-method) for attitude estimation was a method developed by Paul Davenport [12,13] as a compact solution for optimal estimation of the rotation of a body relative to an Inertial frame. Quaternions are widely used because of their efficiency and computational advantages [14,15] over other three-parameter attitude representations, such as Euler angles [16,17]. They are ideal for avoiding singularities, such as gimbal lock, which can occur when using Euler angles during large rotations [18]. Quaternions are also computationally lighter and more stable in dynamic conditions, which makes them popular in real-time applications [19,20]. The integration of a quaternion-based algorithm with the Kalman filter has been widely applied for real-time attitude estimation in aerospace applications [21,22]. Another progression has led to the development of more advanced algorithms, such as Unscented Kalman Filters (UKFs) and particle filters, which demonstrate enhanced performance in handling non-linear systems [23]. Additionally, recent advancements have incorporated deep learning approaches to enhance the pose estimation process. For example, methods that fuse quaternions with convolutional neural networks (CNNs) have demonstrated high levels of accuracy in both orientation and position estimation [24]. These developments have researched uncoupled attitude estimation without integrating the translational dynamics. The need for solutions that integrate the coupled dynamics is essential for many motion-based proximity applications.

This paper generalizes Davenport’s q-method to solve for the relative pose—the coupled state of attitude and translation—for estimation of UAVs with onboard measurements. This paper first provides a comprehensive overview of mathematical preliminaries and Davenport’s q-method for attitude estimation, including a background on the Wahba problem [25] and laying a solid foundation for the subsequent advancements. The development of the quaternion-based approach is explained, highlighting its advantages over traditional methods in terms of simplicity and reduced computational overhead. Following this, the q-method pose estimation model is formulated as a novel, more generalized approach that optimally estimates a body’s position and orientation. This approach requires two sets of measurements for reference points, enabling the calculation of the optimal relative pose from an Inertial frame. This framework eliminates various assumptions inherent to the original technique for spacecraft application and provides a precise method for aircraft and spacecraft pose estimation when given known inertial reference data and body-generated measurements. The intricacies and methodologies here are fully documented. Subsequently, a simulation is constructed to corroborate the practical application of the newly devised pose estimation method in a controlled environment. Numerical methods are employed to solve the newly formed six degrees of freedom (6DoF) estimation equations to minimize the system error. This multifaceted analysis ensures empirical evidence of this effective technique in real-world settings.

By unifying the models for attitude and position estimation, the generalized q-method offers a comprehensive solution for the relative pose estimation problem, bridging the gap between quaternion-based rotation estimation and full pose determination. Through rigorous derivation of the governing equations and models, we provide a cohesive framework that extends the reach of the q-method, setting the stage for its practical application in aerospace systems and beyond. The main contribution is summarized as the development of a generalized q-method for pose estimation. This method is based on formulating the coupled dynamics of 6DoF motion in a singularity-free manner. The generalized q-method was tested using relative pose environments in two different attitude configurations with realistically acquired data and an experimental setup in the SPACE lab. The performance of the method was validated against ground truth data obtained from a Vicon system, which provides sub-millimeter accuracy.

## 2. Mathematical Preliminaries

### 2.1. Coordinate Frame and System Definition

**Figure 1:**
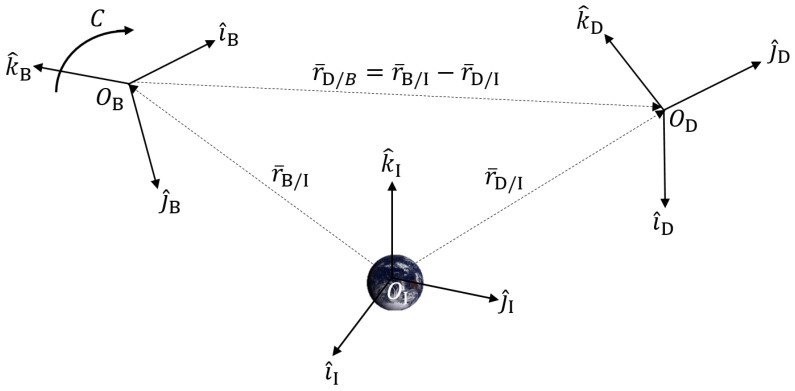
Relative coordinate frame system definition.

Illustrated in Figure 1, there are three frames: the Inertial reference (I) frame, the Body reference (B) frame, and the relative reference (D) frame. The Inertial frame remains fixed in space, while the Body frame is free to move throughout the system, relative to the I frame. The B frame is oriented by some rotation *C* (Direction Cosine Matrix) relative to the I frame. The respective position vectors from the Inertial frame (system origin) are given by ${\bar{r}}_{B/I}$ and ${\bar{r}}_{D/I}$. ${\bar{r}}_{D/B}$ goes to D from B, and can be given with respect to the body-centered frame ${\bar{r}}_{D/B}^{B}$, or the inertial-centered frame ${\bar{r}}_{D/B}^{I}$$∵C{\bar{r}}_{D/B}^{I}={\bar{r}}_{D/B}^{B}$. This relation will be upheld across estimation methods; the upfront definitions are given because individual notation varies, but the fundamentals bind together the varying models with a unifying system. Ultimately, the final goal is to obtain a solution that yields the rotation *C* or *q* and the position of the body with respect to the Inertial frame ${\bar{r}}_{B/I}^{I}$. This general system relation will be interrelated across all forms of this problem.

### 2.2. Wahba’s Least Squares Optimization Problem

In 1965, mathematician Grace Wahba formulated an intuitive way to describe a rotation between two coordinate systems [25]. This method built the problem as a least squares optimization problem to minimize the error between the known relative reference position vector in the Inertial frame ${\bar{r}}_{D/B}^{I}$ and the measurements in the B frame ${\bar{r}}_{D/B}^{B}$. This optimization problem can then lead to the optimal estimate of the rotation matrix *C* that satisfies the relation between the two frames $C{\bar{r}}_{D/B}^{I}={\bar{r}}_{D/B}^{B}$. For ease, we will designate $\hat{r}$ as the unit vector for ${\bar{r}}_{D/B}^{I}$ and $\hat{b}$ as the unit vector for ${\bar{r}}_{D/B}^{B}$ used in this model. Also, this model was derived for spacecraft attitude estimation purposes and finds use in [26] with the simplification $\bar{r}={\bar{r}}_{D/I}^{I}$≈${\bar{r}}_{D/B}^{I}$ (Referencing Figure 1). This assumption can be made when ${\bar{r}}_{D/I}^{I}$>>${\bar{r}}_{B/I}^{I}$ where the relative distance of a celestial body for D reference measurements (e.g., star catalog) is far greater than the distance of a satellite body to the earth.

Now, the relation for each reference vector $C{\hat{r}}_{i}={\hat{b}}_{i}$ is applicable in an ideal world where body-frame measurements are without any error. In reality, all *N* measurements ${\hat{b}}_{i}$ will posses some form of innate sensor error, noise, or uncertainty. The error term for each measurement can be condensed into $\delta_{i}$ and gives the relation ${\hat{b}}_{i}=C{\hat{r}}_{i}+\delta_{i}$.

This can then be formulated as a least squares problem to find the solution for *C* to minimize the error $\delta_{i}$ in the system shown in Equation (1). Furthermore, by expanding the least squares it is converted into a minimization problem for L(C) with Equation (2), and solving for the optimal direction cosine matrix *C*.

(1) $$\delta_{i}^{2}={∥{\hat{b}}_{i}-C{\hat{r}}_{i}∥}^{2}={\left({\hat{b}}_{i}-C{\hat{r}}_{i}\right)}^{T}\left({\hat{b}}_{i}-C{\hat{r}}_{i}\right)$$

(2) $$L\left(C\right)=\frac{1}{2}\sum_{i=1}^{n}\alpha_{i}\delta_{i}^{2}=\;\frac{1}{2}\sum_{i=1}^{n}\alpha_{i}\left({\hat{b}}_{i}^{T}{\hat{b}}_{i}+\;{\hat{r}}_{i}^{T}C^{T}C{\hat{r}}_{i}-{\hat{r}}_{i}^{T}C^{T}{\hat{b}}_{i}-{\hat{b}}_{i}^{T}C{\hat{r}}_{i}\right)$$

Given that L(C)=min, it can also be modeled as a maximization problem where G(C)=max. The term $\alpha_{i}$ is a set of non-negative weights for each observation; $\alpha_{i}$ may remain unweighted with a scalar value of one if desired or uncertain. Through some algebraic simplification, the following equation is obtained:

(3) $$G\left(C\right)=\;\sum_{i=1}^{n}\alpha_{i}{\hat{b}}_{i}^{T}C{\hat{r}}_{i}=1-L\left(C\right)=\;max$$

This leads to the one final equation in simplified form, maximizing the trace of the direction cosine matrix *C*, Equation (4), and the summation matrix *B*, Equation (5).

(4) $$G\left(C\right)=\;\mathrm{tr}\left[CB^{T}\right]=\;max$$

(5) $$B=\;\sum_{i=1}^{n}\alpha_{i}{\hat{b}}_{i}{\hat{r}}_{i}^{T}$$

This forms the basis of Wahba’s problem for optimal attitude estimation. The objective is to solve for a direction cosine matrix *C* that maximizes the function G(C). However, it is not always the easiest to solve for *C* directly—although there have since been solutions developed to do so [27]. The formalized solution to obtain the solution using the quaternion is given in the following sections.

### 2.3. Functional Concepts for Quaternions

The quaternion [28] *q* is an application of complex numbers on a Clifford algebra in $R^{4}$ defined as the following:

(6) $$q=q_{0}+q_{1}i+q_{2}j+q_{3}k$$

The group of quaternions as defined by Hamilton in 1843 [29] utilizes the imaginary units that follow the definition $i^{2}=j^{2}=k^{2}=ijk=-1$ and $\left\{q_{0},q_{1},q_{2},q_{3}\in R\right\}$. It is also common to represent the quaternion as two components, the vector component (i,j, and k) and the scalar component (denoted by $q_{0}$). The purpose of the scalar component is to provide an additional, redundant parameter that keeps the quaternion fully defined in the event that a singularity may occur. This keeps the quaternion singularity free. Another way of thinking of it is thus:

(7) $$q=(q_{0},\bar{q})$$

Note that $q_{0}=\cos\left(\frac{\phi }{2}\right)\in R$ and $\bar{q}={\left[q_{1},q_{2},q_{3}\right]}^{T}=\bar{e}\sin\left(\frac{\phi }{2}\right),\in R^{3}$. Here, $\bar{e}$ is the principal axis unit vector [i,j,k] and ϕ is the principal angle for attitude and rotation representation purposes. Additionally, the quaternion may sometimes be defined with the scalar component last as $q_{4}$. However, for the purpose of this paper, the quaternion will always use the scalar component as the first element $q_{0}$. In practice, the scalar component tells the angle of rotation, and the normalized vector component provides the direction of the rotation axis.

A quaternion used for attitude representation is a unit quaternion (also called rotation quaternion) with norm ∥q∥=1 and satisfies the condition $q^{T}q=1$—similarly to how a direction cosine matrix (DCM) possesses its orthogonal property such that $CC^{T}=I_{3\times 3}$. The norm constraint is the additional parameter that fully constrains the quaternion in the event of an angular singularity. A quaternion describing the orientation of the X frame from the Y frame $\left(q_{X/Y}\right)$ satisfies the condition ${\left(q_{Y/X}\right)}^{*}\left(q_{Y/X}\right)=\left(q_{Y/X}\right){\left(q_{Y/X}\right)}^{*}=1_{q}$, where $1_{q}≜\left(1,{\bar{0}}_{3\times 1}\right)$. See Table 1 for operation definitions.

Due to the quaternion being defined in such a way that it is constructed with a scalar component and a vector component, ordinary linear algebra operations may not apply to the quaternion as they would a typical vector. As such, Table 1 [30] summarizes the operations that are implemented when working with quaternion algebra. For example, the distinction must be made that ${\left(q_{Y/X}\right)}^{*}\left(q_{Y/X}\right)$ is the quaternion multiplied with its conjugate using the quaternion operations, while $q^{T}q$ is to be taken as the traditional 4 × 1 column vector ordinarily multiplied by its transpose.

Three-dimensional vectors may also be interpreted as special cases of quaternions. This allows for combined use of quaternions along with vectors in a three-dimensional space for dynamics governing equations. Redefining a vector $\bar{s}$ such that $\bar{s}\in R^{3}$ is in the form of a quaternion is carried out as shown below.

(8) $$s_{q}=\left(s_{0},\bar{s}\right)\;\;\;\;\mathrm{with}\;\;s_{0}=0$$

Thus, the distinction is formed to differentiate the quaternion itself used for attitude representation, and variables in ‘quaternion’ form. Consider that when a vector $\bar{s}$ is converted into quaternion form, it will not be a unit quaternion. Lastly, the quaternion has explicit applications for changing reference frames—both in general and also for variables in quaternion format. The change of reference frame for a vector in quaternion form from the X frame to the Y frame is achieved via the following:

(9) $$s_{q}^{Y}=q_{Y/X}^{*}s_{q}^{X}q_{Y/X}$$

### 2.4. Davenport’s q-Method for Attitude Estimation

A solution for this estimation problem may be obtained by substituting the quaternion in place of the direction cosine matrix. The equation below is an identity for a direction cosine matrix as a function of *q*. Here, $q_{0}$ is the quaternion scalar component, $\bar{q}$ is the quaternion vector component, and $\left[\bar{q}\times \right]$ is the skew-symmetric matrix of $\bar{q}$.

(10) $$C\left(q\right)=\left(q_{0}^{2}-{\bar{q}}^{T}\bar{q}\right)I_{3\times 3}+2\bar{q}{\bar{q}}^{T}-2q_{0}\left[\bar{q}\times \right]$$

Through some manipulation, this optimization problem can be parameterized in terms of *q*. By substituting Equation (10) into G(q) for Equation (4) and isolating the quaternion, the system objective function G(q) can be put into a compact format of $q^{T}Kq$. *K* is a 4 × 4 matrix, and this provides a quadratic form which will allow for easy minimization [31] (or maximization, in this case).

(11) $$\begin{array}{cc} G\left(q\right) & ={\left\{q_{0}\;\;\;\bar{q}\right\}}^{T}\left[\;\begin{array}{cc} tr\left(B\right) & \sum_{i=1}^{n}\alpha_{i}\left[{\hat{b}}_{i}\times \right]{\hat{r}}_{i}\\ {\left(\sum_{i=1}^{n}\alpha_{i}\left[{\hat{b}}_{i}\times \right]{\hat{r}}_{i}\right)}^{T} & \left(B+B^{T}-tr\left(B\right)I_{3\times 3}\right) \end{array}\right]\left\{\begin{array}{c} q_{0}\\ \bar{q} \end{array}\right\}\\ & =q^{T}Kq \end{array}$$

A constraint $q^{T}q$ = 1 is then added to solve for the nontrivial solution of the optimization problem, as shown in Equation (12). This provides the optimal quaternion $q_{opt}$ that defines the rotation between two frames. Afterwards, differentiating with respect to *q* and equating *q* = 0 solves for the maximum value of this quadratic form, shown in Equation (13).

(12) $$G^{*}\left(q\right)=q^{T}Kq-\lambda \left(q^{T}q-1\right)=max$$

(13) $$\frac{dG^{*}\left(q\right)}{dq}=2Kq-2\lambda q=0\;$$

(14) $$Kq_{opt}=\lambda_{max}q_{opt}$$

This yields a very straightforward solution where the optimal quaternion $q_{opt}$ is exactly the eigenvector that corresponds to the maximum real eigenvalue $\lambda_{max}$ for matrix *K*. Therefore, following this procedure provides $q_{opt}$ to minimize the error in the attitude estimation system via the *K* matrix. This involves using summations of the *B* matrix—constructed with measurement vectors ${\hat{b}}_{i}$ and known reference direction vectors ${\hat{r}}_{i}$. Given the relation for Equation (10), the solution will provide the rotation matrix *C* that describes the rotation transformation from the Inertial frame to the Body frame.

For awareness, there are some nuances that can be added to the q-method attitude estimation process, the first being that ${\hat{b}}_{i}$ and ${\hat{r}}_{i}$ are not required to be directional unit vectors. Both ${\bar{b}}_{i}$ and ${\bar{r}}_{i}$ can be positional vectors instead. The results for attitude estimation are identical; the exact derivation of the Wahba problem and q-method would differ slightly due to a lack of simplification that comes with the unit vectors, but the core process and equations will remain the same. In practice, unit directional vectors are more commonly used [32] because knowledge of the precise distance for a reference body can be difficult, especially for satellite applications.

With the above assumptions in play, Davenport’s q-method yields great results. However, we can develop a more generalized model that will continue to be applicable for satellite applications, but will not be limited by approximations or simplifications. The following section will expand on this knowledge to incorporate position estimation in tandem with attitude estimation to formulate a robust pose estimation method.

## 3. q-Method Pose Estimation Derivation

**Figure 2:**
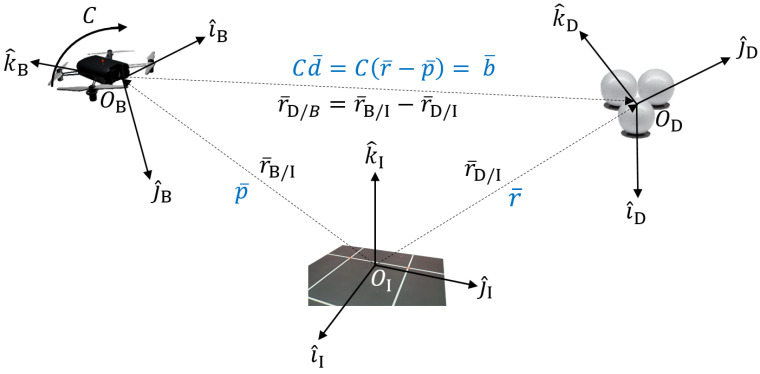
Definition of q-method pose estimation system.

This section will now shift focus onto the newly developed q-method for pose estimation. Prior use of the q-method focused on spacecraft applications for attitude estimation, whereas this more generalized approach eliminates both the unit vector direction and $\bar{r}={\bar{r}}_{D/I}^{I}$≈${\bar{r}}_{D/B}^{I}$ assumptions for the ’known’ reference values of ${\bar{r}}_{i}$. This indeed complicates the problem and makes it more difficult to estimate, but also incorporates inertial position $\bar{p}$ into the estimation; the final model shows promising compatibility for 6DoF drone pose estimation. Figure 2 illustrates how the q-method pose estimation will relate back to the global system definitions in Figure 1, differing from the original q-method. The objective is to estimate the attitude, *C*, as well as the satellite position, $\bar{p}$, in order to estimate the pose of the air vehicle. A list of variables is provided:

- ${\bar{r}}_{B/I}^{I}$—Body position from Inertial Reference (Earth), to be estimated, $\bar{p}$.
- ${\bar{r}}_{D/I}^{I}$—Known reference position from Inertial frame (Earth), $\bar{r}$.
- ${\bar{r}}_{D/B}^{B}$—Measured position vectors in the Body frame, with error, $\bar{b}$.
- ${\bar{r}}_{D/B}^{I}$—Unknown. Essentially requires prior knowledge. If we had this, position estimation would be easy. Let it be known as $\bar{d}$. We will use this to estimate $\bar{p}$ given that $\bar{d}=\bar{r}-\bar{p}$ by following the vector addition for the system definition.

We are able to take advantage of a simple substitution for ${\bar{r}}_{D/B}^{I}$ by virtue of system definition commonality. The q-method takes ${\bar{r}}_{D/B}^{I}=\hat{r}$. For the q-method pose derivation, let ${\bar{r}}_{D/B}^{I}=\bar{d}=\bar{r}-\bar{p}$. The relation for the drone body measurements with error continues as ${\hat{b}}_{i}=C{\hat{d}}_{i}+\delta_{i}$. Substituting this equivalence into Equation (1), a slight modification is made to the original Wahba problem. This straightforward substitution unifies the estimation derivations, allowing for a similar system of equations. Throughout this derivation, all aforementioned variables will be kept identical, including the weighting factor $\alpha_{i}$. Curiously, there exists other research expanding upon the Wahba problem to incorporate position [33,34], but not for the quaternion-based purposes used here.

(15) $$\begin{array}{cc} L\left(C\right)=\delta_{i}^{2} & =∥{\bar{b}}_{i}-C\left({\bar{r}}_{i}-{\bar{p}}_{i}\right){∥}^{2}={\left({\bar{b}}_{i}-C\left({\bar{r}}_{i}-{\bar{p}}_{i}\right)\right)}^{T}\left({\bar{b}}_{i}-C\left({\bar{r}}_{i}-{\bar{p}}_{i}\right)\right)\\ \; & ={\left({\bar{b}}_{i}-C{\bar{d}}_{i}\right)}^{T}\left({\bar{b}}_{i}-C{\bar{d}}_{i}\right) \end{array}$$

Further expanding the minimization cost function L(C,d), and then grouping terms, gives

(16) $$L\left(C,d\right)=\frac{1}{2}\sum_{i=1}^{n}\alpha_{i}\delta_{i}^{2}=\;\frac{1}{2}\sum_{i=1}^{n}\alpha_{i}\left({\bar{b}}_{i}^{T}{\bar{b}}_{i}+\;{\bar{d}}_{i}^{T}C^{T}C{\bar{d}}_{i}-{\bar{d}}_{i}^{T}C^{T}{\bar{b}}_{i}-{\bar{b}}_{i}^{T}C{\bar{d}}_{i}\right)$$

(17) $$L\left(C,d\right)=\;\frac{1}{2}\sum_{i=1}^{n}\alpha_{i}{\bar{b}}_{i}^{T}{\bar{b}}_{i}+\frac{1}{2}\sum_{i=1}^{n}\alpha_{i}{\bar{d}}_{i}^{T}{\bar{d}}_{i}-\sum_{i=1}^{n}\alpha_{i}{\bar{b}}_{i}^{T}C{\bar{d}}_{i}$$

Note that (17) possesses two terms that Equation (3) combines into one, due to unit vector measurements. Equation (3) also converts the problem into a maximization problem for simplicity, but the above form continues with the form $L(C,\bar{d})$ and $L(q,\bar{d})$, opting to keep the third term negative. Also, note the dimensions of *q* (4 × 1) and $\bar{d}$ (3 × 1); the following equations make use of the aforementioned ’quaternionized’ vector format in Equation (8) when required.

The third term also still resembles that in (3), and subsequently will be equivalent to $\mathrm{tr}[C\beta^{T}]$. Like *B* in Equation (5), β is defined as

(18) $$\beta =\;\sum_{i=1}^{n}\alpha_{i}{\bar{b}}_{i}{\bar{d}}_{i}^{T}$$

Similarly, the cost function is put into terms of $L(q,\bar{d})$ using Equation (10). Only the third term is a function of *q* (or *C*), and substituting $\bar{d}$ for $\hat{r}$ allows for the use of (11), as derived by Davenport. The cost function then becomes

(19) $$L\left(q,d\right)=\frac{1}{2}\sum_{i=1}^{n}\alpha_{i}{\bar{b}}_{i}^{T}{\bar{b}}_{i}+\frac{1}{2}\sum_{i=1}^{n}\alpha_{i}{\bar{d}}_{i}^{T}{\bar{d}}_{i}-q^{T}\kappa q$$

where the third term $q^{T}\kappa q$ is expressed as

(20) $$q^{T}\kappa q={\left\{q_{0}\;\;\;\bar{q}\right\}}^{T}\left[\;\begin{array}{cc} \mathrm{tr}\left(\beta \right)\; & \sum_{i=1}^{n}\alpha_{i}\left[{\bar{b}}_{i}\times \right]{\bar{d}}_{i}\\ {\left(\sum_{i=1}^{n}\alpha_{i}\left[{\bar{b}}_{i}\times \right]{\bar{d}}_{i}\right)}^{T} & \left(\beta +\beta^{T}-\mathrm{tr}\left(\beta \right)I_{3\times 3}\right) \end{array}\right]\left\{\begin{array}{c} q_{0}\\ \bar{q} \end{array}\right\}$$

With the cost function f(x) now in terms of the variables $x=[q,\bar{d}]$ of interest, the quaternion norm constraint $q^{T}q=1$ is imposed as an equality constraint g(x). The Lagrangian function [35] is applied to obtain the optimal solution that minimizes the objective function f(x) (minimizing the error), therefore obtaining the optimal solution for *q* and $\bar{d}$. The Lagrangian function is defined as L(x,λ)≡f(x)−λ〈g(x)〉.

(21) $$L\left(q,d,\lambda \right)=\frac{1}{2}\sum_{i=1}^{n}\alpha_{i}{\bar{b}}_{i}^{T}{\bar{b}}_{i}+\frac{1}{2}\sum_{i=1}^{n}\alpha_{i}{\bar{d}}_{i}^{T}{\bar{d}}_{i}-q^{T}\kappa q-\lambda \left(q^{T}q-1\right)$$

The necessary condition is implemented to minimize the system such that $\frac{\partial L}{\partial \bar{d}}=0$ and $\frac{\partial L}{\partial q}=0$. The partial differential equations used to solve the system are shown below.

(22) $$\frac{\partial L}{\partial \bar{d}}=0=\sum_{i=1}^{n}\alpha_{i}{\bar{d}}_{i}-q^{T}\frac{\partial \kappa }{\partial d}q$$

(23) $$\frac{\partial L}{\partial q}=0=2\kappa q-2\lambda q$$

Equations (22) and (23) can now be used to solve for the optimal position estimation ${\bar{p}}^{*}$ and optimal quaternion to describe the rotation $q_{opt}$. $\frac{\partial L}{\partial q}$ fortunately gives a solution comparable to the q-method, making use of κ shown in (20) to find $q_{opt}$ as the eigenvector for the corresponding maximum real eigenvalue. E.g., (22) will substitute ${\bar{d}}_{i}=\left({\bar{r}}_{i}-{\bar{p}}^{*}\right)$ so that $\sum_{i=1}^{n}\alpha_{i}{\bar{d}}_{i}=\sum_{i=1}^{n}\alpha_{i}{\bar{r}}_{i}-\sum_{i=1}^{n}\alpha_{i}{\bar{p}}^{*}$. The end set of equations for q-method pose estimation are hereby formed.

(24) $${\bar{p}}^{*}=\frac{1}{N}\left(\sum_{i=1}^{n}\alpha_{i}{\bar{r}}_{i}-q^{T}\frac{\partial \kappa }{\partial \bar{d}}q\right)$$

(25) $$\kappa q_{opt}=\lambda_{max}q^{*}$$

One last simplification is made due to the term $\frac{\partial \kappa }{\partial \bar{d}}$ within Equation (24). The partial differential of $\kappa_{4\times 4}$ w.r.t. a vector $\bar{d}\in R^{3}$ yields a 4 × 4 × 3 higher-order tensor. The evaluation of this term can be found in a prior publication [36] as well as validation of q-method pose estimation for simulated spacecraft applications. Thus, an intuitive equation for ${\bar{p}}^{*}$ exists by again referencing the system definition and taking a weight average for $\bar{p}$ = ${\bar{r}}_{B/I}^{I}={\bar{r}}_{D/I}^{I}-{\bar{r}}_{D/B}^{I}$. By definition, ${\bar{r}}_{D/B}^{I}=q_{I/B}{\bar{r}}_{D/B}^{B}q_{I/B}^{*}=q_{B/I}^{*}{\bar{b}}_{i}q_{B/I}$, where $q^{*}$ is the quaternion conjugate.

(26) $${\bar{p}}^{*}=\frac{1}{N}\left(\sum_{i=1}^{n}\alpha_{i}{\bar{r}}_{i}-q\langle \sum_{i=1}^{n}\alpha_{i}{\bar{b}}_{i}\rangle q^{*}\right)$$

To summarize, the goal of q-method pose estimation is to find ${\bar{p}}^{*}$ and $q_{opt}$. In order to do so, one must first formulate κ, making use of β, which makes use of $\bar{d}$. If one of the optimal values (${\bar{p}}^{*}$ or $q_{opt}$) is known, the other can then easily be calculated. However, a solution can be found using any valid numerical solving method. The next sections will investigate the results using a numerical solving scheme in parallel with physical sensors in a controlled lab environment.

## 4. Experimental Setup and Methodology

### 4.1. Experimental Overview and Setup

**Figure 3:**
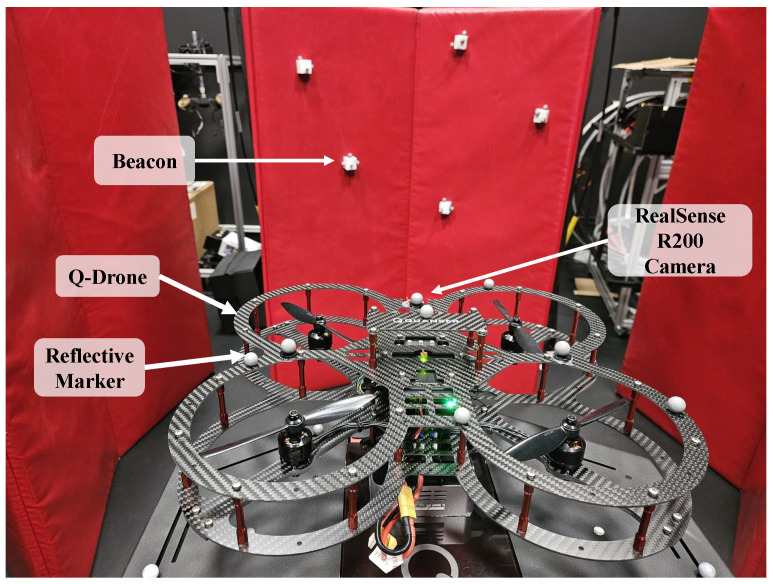
Quanser drone and five reference beacons.

**Figure 4:**
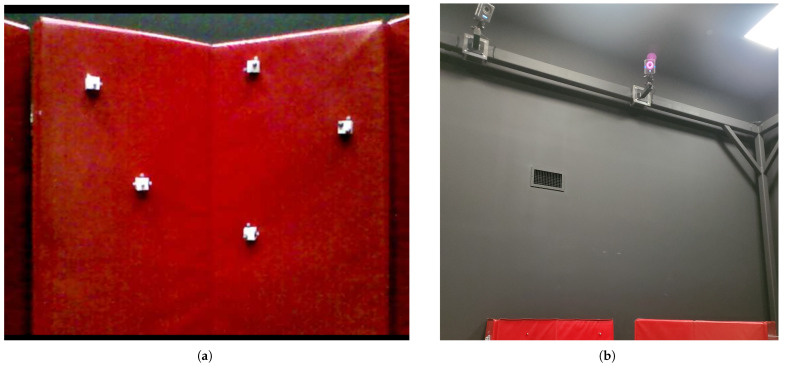
(**a**) RGB imagery from drone camera. (**b**) Partial Vicon system within SDSU SPACE lab.

**Figure 5:**
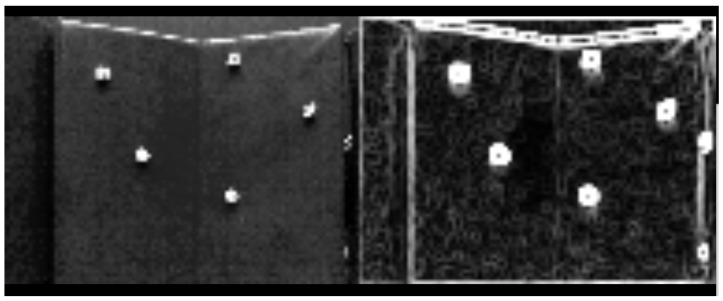
Beacon detection and location definitions.

To further verify the functionality of the system of equations, the q-method pose estimation is incorporated with onboard measurements obtained from drone-camera experiments. Figure 3 illustrates how a Quanser drone used an Intel RealSense (R200) camera to measure the locations of five objects/beacons in 3D space. The beacons are placed at varying locations and depths upon a curved surface. The camera’s depth-sensing feature was used to determine the location of each beacon in space relative to the camera/drone frame. Camera detection of each beacon is shown in Figure 4 and Figure 5. These are used to provide positional estimates in a pictured frame, and then converted into the body-centered drone frame.

To validate correct estimates, the results were compared against true reference data. Vicon’s motion capture system defined the locations of the five beacons as well as the drone. The Vicon system consists of eight high-resolution cameras that capture moving objects at a rate of 120 Hz. Each beacon and drone were defined as rigid bodies (objects) in three dimensions using reflective markers. Figure 4 shows two of the eight Vicon cameras mounted across the lab and test setup, forming the Inertial frame. This provides a full overview of the hardware used for the experimental setup across the Body and Inertial frames.

**Figure 6:**
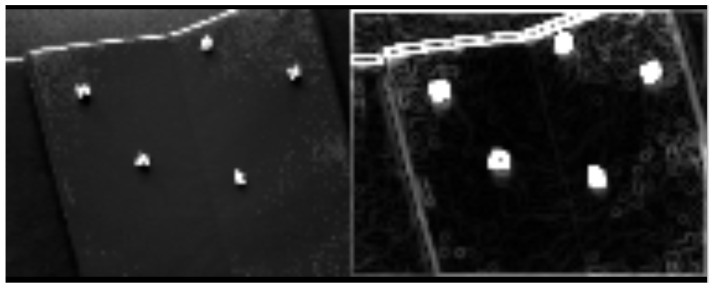
Reference beacons measured with $15^{\circ }$ tilted drone.

To test the method for orientation and position estimation in a physical setting, the drone operated on a static mount while detecting the beacons to obtain reference measurements. The first experiment used a stabilized, nominally oriented (no-tilt) drone to measure beacons, as shown in Figure 5 with the drone camera. A second experiment was performed where the drone was statically tilted at a roll angle of −15 degrees in the Body frame, as shown in the Figure 6, also with the onboard camera.

Two corrections to the experimental setup were required while post-processing data. First, the camera position placed on the drone needed to be taken into account, as it was +15 cm ahead of the true Vicon-defined drone centroid. This fix was performed by adding a +15 cm bias to the Vicon positional truth data during the pose estimation analysis and validation. Second, the camera measurements contained a constant bias outside of the camera position that needed to be considered. This was achieved by performing a static calibration in the same position as the experiment for both drone orientations. Afterwards, the mean bias of the camera for each individual reference marker was subtracted. Further details will be provided in the following section after the inputs.

### 4.2. Initial Conditions and Inputs

**Figure 7:**
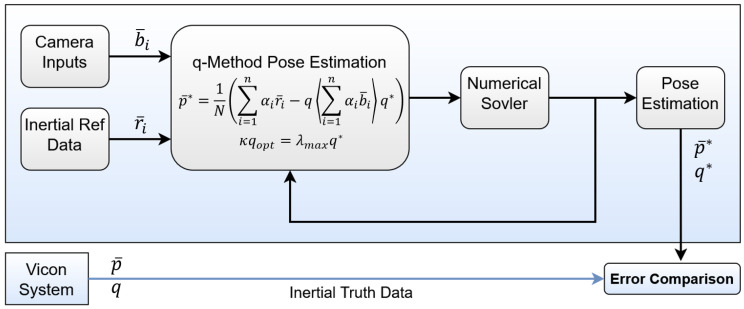
Procedural validation for q-method pose estimation.

**Figure 8:**
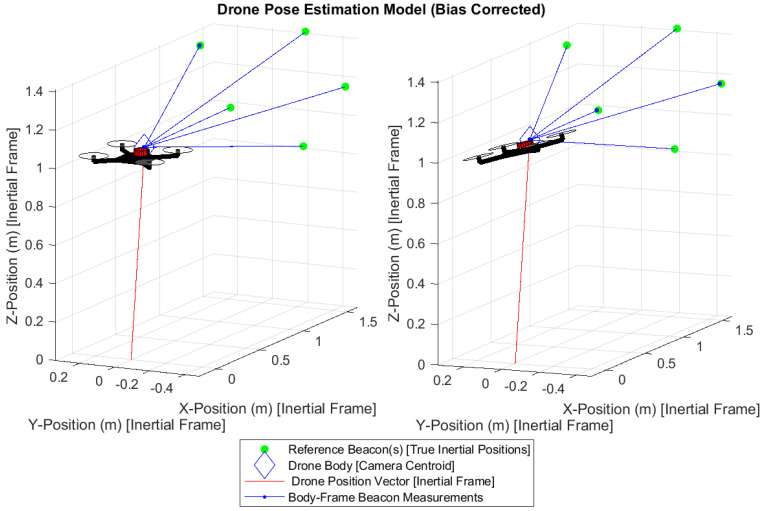
Experimental visualization. [Left: $0^{\circ }$ roll. Right: $-15^{\circ }$ roll].

The two experiments were performed and the resulting output data were fed into MATLAB 2018a for post-processing, algorithm implementation, and analysis. Figure 7 provides an illustration of or pseudo-code for the validation process for both configurations. Figure 8 provides a visual representation of the true data within MATLAB using the Vicon system. A correction factor of +15 cm is added to account for the position of the camera on the drone with respect to the Body frame. This offset can be seen in the figure, as the camera centroid is offset from the drone body itself. Additionally, several hundred measurements were taken to characterize the average measurement bias of the camera sensor and correct the input reference measurements. The estimation algorithm and results come from a single instantaneous state—one each for the $0^{\circ }$ roll and −$15^{\circ }$ roll.

The pose estimation using the previously outlined system of equations was accomplished with the aid of the built-in MATLAB numerical equation solver fsolve for this multi-variable system. The function uses a Trust-Region Dogleg method to solve for the optimal solution that minimizes the residual error of the system. The following is the exact input to calculate the residual f(x) used within the solver based on Equations (25) and (26).

(27) $$f\left(x\right)=0=\left[\begin{array}{c} \sum_{i=1}^{n}\alpha_{i}\left({\bar{r}}_{i}-{\bar{p}}^{*}\right)-q\langle \sum_{i=1}^{n}\alpha_{i}{\bar{b}}_{i}\rangle q^{*}\\ \left(2\kappa q-2\lambda q\right)/10 \end{array}\right]$$

The second row of f(x) is multiplied by $10^{-1}$ in order to better condition the system. Equating both sides of the equation to 0, this multiplication scalar does not make a difference to the solution. λ is included for the purpose of solving the system numerically. Thus, $f(\bar{p},q,\lambda )=0$ where $x=[\bar{p},q,\lambda ]$ to minimize the system.

An initial guess $x_{0}$ is required to iteratively solve the function via numerical methods. As such, a nearby initial guess is arbitrarily selected for the quaternion $q_{0}$, position $p_{0}$, and λ of the system. For this experiment, let $p_{0}=\left[0,0,1\right]$ m, $q_{0}=\left[1;0;0;0\right]$, and $\lambda_{0}=\Sigma \bar{b_{i}}\;{\bar{b_{i}}}^{T}$. This formulation for $\lambda_{0}$ is obtained through empirical observations of what generally works well while using this estimation model in conjunction with a numerical equation solver.

Table 2 provides the value of each parameter to initialize the system. The results generated hereafter make use of these provided inputs. Given N reference points for known reference vectors ${\bar{r}}_{D/I}^{I}$ and N body measurements ${\bar{b}}_{i}$ with measurement error $\sigma_{i}$, the objective is to estimate ${\bar{p}}^{*}$ and $q_{opt}$ to be as close to the true values ${\bar{r}}_{B/I}^{I}$ and $q_{B/I}$ as possible.

Measurement errors and correction factors across each roll condition are all provided in Table 2 for transparency. Starting with the reference measurement error $\sigma_{i}$, a scalar percent error value is given for each on-board measurement of each reference vector in the Body frame. $\sigma_{i}$ is computed using the experimental and true positional values of the reference beacons with respect to the camera given as $|{\bar{r}}_{D/B}^{B}-\bar{b_{i}}\left|/\right|{\bar{r}}_{D/B}^{B}|\times 100\%$. Mean measurement error $∥\sigma_{i}{∥}^{2}$ is the average error across all N measurements used in this procedure. This measurement error, while missing directional value, is moreso a simplified method to partially quantify the error in the system when comparing the accompanying algorithm results.

In addition, the average sensor error $\bar{\sigma }$ is the average error across time (700 data points) of the camera sensor for each reference beacon. A static collection of data identical to $\bar{b_{i}}$ is performed to verify that the snapshot state obtained in $\bar{b_{i}}$ is not drastically different from the average of the camera’s ordinary noisy measurements. The same static collection of data was also used to detect the sensor’s average experimental bias $\delta {\bar{r}}_{D/B}^{B}$ to correct the raw measurement vectors ${\bar{b_{i}}}^{†}$. Thus, the bias correction is made such that $\bar{b_{i}}={\bar{b_{i}}}^{†}+\delta {\bar{r}}_{D/B}^{B}$. Information for methods and error are disclosed, but procedures for absolute correction and minimization of sensor error are intentionally not incorporated here due to the desire to understand estimation performance with some degree of measurement error.

## 5. Results

**Figure 9:**
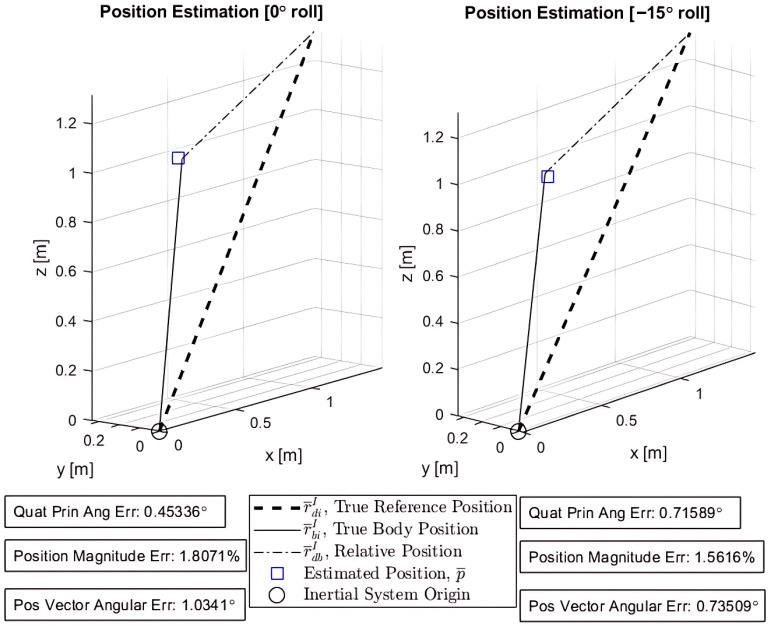
Pose estimation results.

The generalized q-method pose estimation formulation was able to successfully estimate both the position $\bar{p}$ and orientation $q_{opt}$ of the drone for both attitude configurations. Both $\bar{p}$ and $q_{opt}$ are the optimal values for the pose that minimizes the error of the system given the drone camera measurements with sensor noise. Figure 9 is designed to mirror the system definition (Figure 2). It overlays the estimated position $\bar{p}$ onto the true body position vector ${\bar{r}}_{B/I}^{I}$. Here, all inertial and truth data obtained from the Vicon system are used to validate the results of the pose estimation. The inertial system origin is the center floor of the laboratory in which the experiment is performed. True reference position vectors ${\bar{r}}_{D/I}^{I}$ are essential prior-knowledge of the Inertial frame or system, or wherever the drone intends to operate—also obtained by Vicon.

The position error (∼1.8% and 1.56%) is calculated as $∥{\bar{r}}_{B/I}^{I}-{\bar{p}}^{*}∥/∥{\bar{r}}_{B/I}^{I}∥$, and the position angular error is defined as $\cos^{-1}\left(\left({\bar{r}}_{B/I}^{I}\;\cdot \;{\bar{p}}^{*}\right)/∥{\bar{r}}_{B/I}^{I}\;\cdot \;{\bar{p}}^{*}∥\right)$ to represent the angular error between the true vector and estimated position. The quaternion attitude error is not as intuitive to represent, but is found by the principal angle ϕ within the quaternion product $\delta q=q_{B/I}q_{opt}^{*}$ [15] where $q_{opt}^{*}$ is the conjugate of the estimated quaternion. The equation for ϕ is given by $\phi =2\cos^{-1}\left(q_{0}\right)$ from the prior quaternion definition. Observe that the quaternion error (principal angle error, δϕ) for both test cases is ∼0.453°and 0.716° respectively, between the true and estimated quaternion. The small positional error and small quaternion error demonstrate the reliability and accuracy of the q-method for pose estimation despite the relatively few reference measurements.

**Figure 10:**
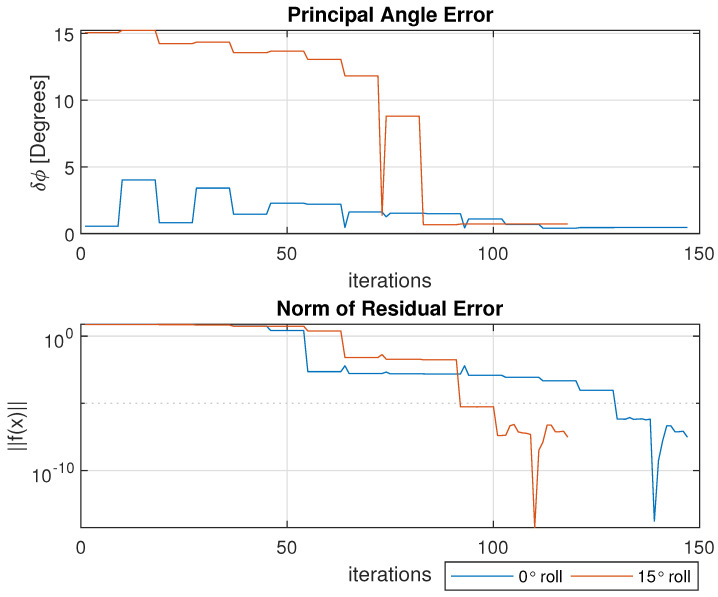
Principal angle error between true and estimated quaternions (**Top**) and norm of residual error for numerical solver (**Bottom**) across iterations.

**Figure 11:**
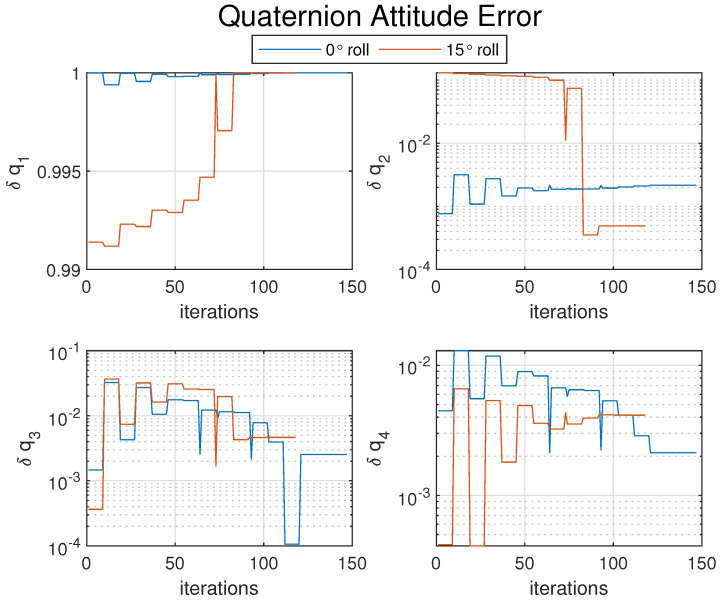
Attitude error between true and estimated quaternion elements [$\delta q_{1},\delta q_{2},\delta q_{3},\delta q_{4}$] across iterations for numerical solver.

**Figure 12:**
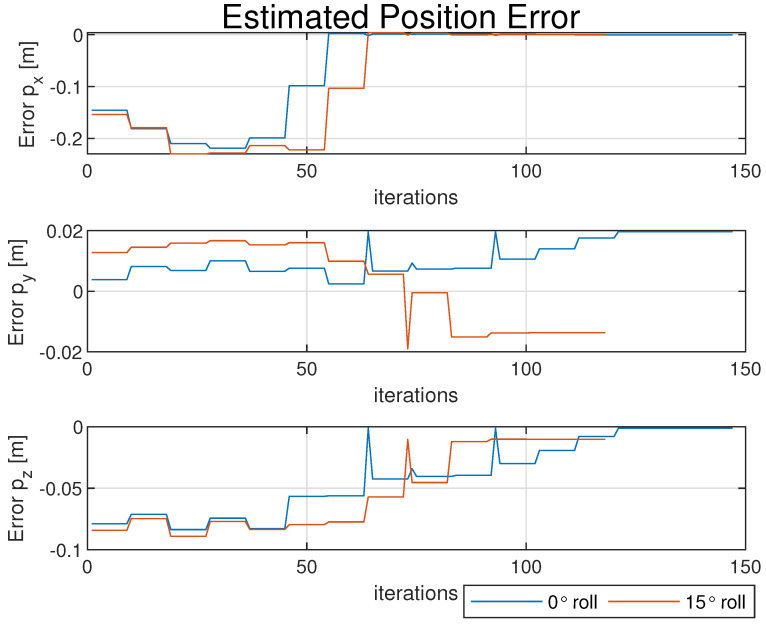
Error for estimated position elements [$p_{x},p_{y},p_{z}$] across iterations for numerical solver.

The remaining figures detail the results of interest across each iteration of the numerical solver until convergence is reached. Iterative results for both the 0° roll and −15° roll cases are overlaid within Figure 10, Figure 11 and Figure 12 for ease of presentation. Figure 10 displays the principal angle error δϕ for an understanding of the aggregate attitude error. The bottom plot of the same figure shows the norm of the residual error ∥f(x)∥ as the function fsolve converges to a stopping condition.

Figure 11 itemizes each element of the attitude error $\left(\delta q_{1},\delta q_{2},\delta q_{3},\delta q_{4}\right)$ between the true quaternion $q_{B/I}$ and the estimated quaternion $q_{opt}$. Lastly, Figure 12 shows the error for each element between the estimated and true positions. The deltas are not expected to be exactly zero; the expectation is not to find the exact true value, but rather to find the optimal estimation that minimizes the error of the system (introduced by the noisy measurements). Further performance enhancements could be made with the addition of more reference points. For an estimation model like this, the enhancements will directly lead to improved performance.

All figures and results presented show the advantage of the pose estimation q-method in its ability to solve for the state of the system with minimal error. In each instance, the numerical equation solver concluded its convergence and obtained a sufficient solution for the UAV. When compared to the inertial, Vicon truth data, the outcome for the predicted position and orientation is greatly comparable despite the error embedded in the attached camera measurement reference vectors.

### Method Comparison and Evaluation

The generalization of the q-method focuses on adopting a solution to estimate both orientation and position simultaneously. The main aim of this paper is not to assess estimation performance or related metrics. Since the proposed algorithm solves the algebraic equation using an eigenvalue approach, it is not entirely fair to compare it with other methods that may involve additional computational steps beyond the scope of this approach. Nevertheless, tests were conducted to compare this method with the q-method (for orientation only) and the modified Optimal Linear Attitude Estimator, OLAE (for orientation and position) [37], showing nearly identical performance results. The same sensor data and initial conditions were used for the comparison.

OLAE is a single-point real-time estimator that utilizes the Rodrigues (or Gibbs) vector, a minimal-element attitude parameterization. The optimality criterion, which differs from Wahba’s constrained criterion, is strictly quadratic and unconstrained. This method estimates both position and orientation based on vector observations obtained through vision-based camera technology. The generalized q-method also estimates the translation vector with the same accuracy and rate. Table 3 summarizes the results.

The results show that both the generalized q-method and OLEA perform similarly in terms of position and orientation errors. At 0° roll angle, OLEA has a slightly smaller position error but a slightly higher orientation error. At 15° roll angle, the position errors for both methods are nearly identical, with OLEA having a marginally larger error. Both methods show an increase in orientation error at 15°, with the generalized q-method maintaining a slight advantage in orientation accuracy. Overall, the differences are small, with both methods offering comparable performance depending on the roll angle.

## 6. Conclusions

The q-method for pose estimation was designed to take the body-frame positional reference measurements and compare them to relative, known positions from an Inertial frame. Doing this, both the orientation and position of the body may be estimated to minimize the error introduced into the system via the measurements. This paper first provided a summary of Davenport’s q-method for attitude estimation and then built on the existing model, while also acknowledging the assumptions and differences that went into the original model. The pose estimation model presented here went to great lengths to keep a consistent system relation between the Inertial, Body, and reference frames in a way that mirrors the q-method. It is via this common system definition that the pose estimation equations were derived by mirroring the original q-method derivation. From this newly developed model, the pose estimation results proved to provide a very accurate solution with relatively simple equations. The computational cost for the numerical methods could be a limitation of this method, but further work could be performed to develop closed-form solutions for this system of equations. Nevertheless, with ample processing power and computing speed, any conventional numerical method used in conjunction with the q-method for pose estimation will provide reliable results.

While results within this paper are promising, the experiment performed was for a single steady-state estimation. Additional estimation approaches like these offer an extra layer of redundancy and dependability for any autonomous aircraft or spacecraft. Further integration into a dynamic system would be required with parallel use of filters, GPS, dynamic models, and/or accelerometer and gyroscopic sensors. Any hardware or communication failures for these flight-essential features would require backup methods for state estimation; the generalized q-method pose estimation provides a backup measure preventing loss of the vehicle, provided prior environmental reference knowledge is utilized.

The estimation error itself is determined by the optimality of the Wahba problem, which is formulated based on the measurement noise. This paper does not aim to reduce the estimation error further, but rather focuses on generalizing the classical q-method to provide pose estimation solutions for coupled vehicle dynamics. Certain validation steps of this pose estimation method were omitted due to redundancy, but additional validation was performed to verify equivalency between the generalized q-method and the classical q-method. Noise reduction and elimination can be fourthly investigated according to the estimation tolerance defined by the problem itself. Preceding work has also previously been performed as part of the validation of the q-method pose estimation by investigating error sensitivity to initial conditions and numerical convergence through a Monte Carlo analysis [36].

Unlike Davenport’s q-method, which will often make the far-away star assumption and use a star-catalogue with respect to earth, this pose estimation method generalizes the model to be more mathematically intuitive for general applications. The pose estimation model is unrestricted to just satellite attitude estimation, as demonstrated in this paper using a drone/UAV. This itself is a benefit. In addition, if the position is already known, these equations will function identically to the q-method. In essence, the integration of pose estimation methodologies with the q-method represents a significant advancement, promising more robust and adaptable solutions for spatial perception and analysis across various domains.

## Figures and Tables

**Table 1:** Quaternion operations.

Operation	Definition
Addition	$a+b=(a_{0}+b_{0},\bar{a}+\bar{b})$
Scalar multiplication	$\lambda a=(\lambda a_{0},\lambda \bar{a})$
Multiplication	$ab=(a_{0}b_{0}-\bar{a}\;\cdot \;\bar{b},a_{0}\bar{b}+b_{0}\bar{a}+\bar{a}\times \bar{b})$
Conjugate	$a^{*}=\left(a_{0},-\bar{a}\right)$
Dot product	$a\;\cdot \;b=(a_{0}b_{0}+\bar{a}\;\cdot \;\bar{b},0_{3\times 1})$
Cross product	$a\times b=(0,a_{0}\bar{b}+b_{0}\bar{a}+\bar{a}\times \bar{b})$
Norm	$||a||=\sqrt{a\;\cdot \;a}$

**Table 2:** Simulation initial condition inputs.

Parameter	Sym.	Unit	Input 0° Roll	Input −15° Roll
Num. Ref. Measurements	*N*	—	5	5
True Orientation	$q_{B/I}$	—	[0.9999; 0.0007; −0.0014; −0.0044]	[0.9913; −0.1309; 0.0003; 0.0004]
True Position	${\bar{r}}_{B/I}^{I}$	[m]	[0.1454, −0.0038, 1.0788]	[0.1539, −0.0127, 1.0842]
Ref. Measurement Error	$\sigma_{i}$	[%]	[0.28, 0.29, 0.23, 0.05, 0.40]	[0.63, 0.64, 0.01, 0.27, 0.36]
Mean Measurement Error	$∥\sigma_{i}{∥}^{2}$	[%]	0.2487	0.3809
Average Sensor Error	$\bar{\sigma }$	[%]	[0.25, 0.24, 0.30, 0.33, 0.25]	[0.29, 0.40, 0.31, 0.42, 0.22]
Measurement Vectors (w/Error)	$\bar{b_{i}}$	[m]	$\left(\begin{array}{ccc} 1.317 & 0.326 & 0.241\\ 1.390 & 0.186 & -0.080\\ 1.472 & -0.201 & 0.336\\ 1.339 & -0.490 & 0.102\\ 1.464 & -0.192 & -0.261 \end{array}\right)$	$\left(\begin{array}{ccc} 1.327 & 0.276 & 0.312\\ 1.367 & 0.224 & -0.036\\ 1.465 & -0.252 & 0.267\\ 1.459 & -0.091 & -0.307\\ 1.324 & -0.470 & -0.032 \end{array}\right)$
True Reference Positions	${\bar{r}}_{D/I}^{I}$	[m]	$\left(\begin{array}{ccc} 1.467 & 0.335 & 1.316\\ 1.530 & 0.194 & 0.994\\ 1.616 & -0.190 & 1.411\\ 1.606 & -0.1821 & 0.814\\ 1.485 & -0.481 & 1.178 \end{array}\right)$	$\left(\begin{array}{ccc} 1.469 & 0.333 & 1.316\\ 1.533 & 0.193 & 0.993\\ 1.618 & -0.188 & 1.409\\ 1.608 & -0.180 & 0.812\\ 1.485 & -0.478 & 1.177 \end{array}\right)$
Raw Measurements	${\bar{b_{i}}}^{†}$	[m]	$\left(\begin{array}{ccc} 1.416 & 0.131 & 0.241\\ 1.507 & 0.013 & -0.089\\ 1.495 & -0.279 & 0.315\\ 1.495 & -0.274 & -0.276\\ 1.355 & -0.483 & 0.084 \end{array}\right)$	$\left(\begin{array}{ccc} 1.404 & 0.173 & 0.142\\ 1.507 & -0.008 & -0.140\\ 1.548 & -0.171 & 0.353\\ 1.478 & -0.314 & 0.213\\ 1.363 & -0.456 & -0.198 \end{array}\right)$
Camera Correction Factor	$\delta {\bar{r}}_{D/B}^{B}$	[m]	$\left(\begin{array}{ccc} -0.099 & 0.196 & 0.000\\ -0.117 & 0.173 & 0.009\\ -0.023 & 0.078 & 0.021\\ -0.156 & -0.216 & 0.378\\ 0.109 & 0.291 & -0.346 \end{array}\right)$	$\left(\begin{array}{ccc} -0.077 & 0.103 & 0.170\\ -0.140 & 0.232 & 0.104\\ -0.083 & -0.081 & -0.087\\ -0.019 & 0.222 & -0.520\\ -0.038 & -0.014 & 0.167 \end{array}\right)$
Weight Parameter	α	—	1.0	1.0

**Table 3:** Comparison of error results for different roll angles.

Roll Angle	Estimation Method	Position Error ∥δp∥[m]	Angle Error δϕ [deg]
0°	q-Method Pose	0.01965	0.4493
	OLEA	0.01898	0.4544
15°	q-Method Pose	0.01708	0.7159
	OLEA	0.01720	0.7202

## Data Availability

The original contributions presented in this study are included in the article material. Further inquiries can be directed to the corresponding author.
